# Stratification system with tumor-associated macrophages for predicting prognostic and therapeutic implications in clear cell renal cell carcinoma

**DOI:** 10.1016/j.jncc.2024.12.009

**Published:** 2026-04-24

**Authors:** Jiahe Lu, Shiqi Ye, Zhongyuan Wang, Yunhong Zhou, Aihetaimujiang Anwaier, Siqi Zhou, Kun Chang, Hailiang Zhang, Dingwei Ye, Wenhao Xu

**Affiliations:** a Department of Urology, Fudan University Shanghai Cancer Center; Department of Oncology, Shanghai Medical College, Fudan University, Shanghai, China; b Shanghai Genitourinary Cancer Institute, Shanghai, China; c Department of Urology, Ruijin Hospital, Shanghai Jiao Tong University School of Medicine, 200025, Shanghai, China; d Department of Radiology, Fudan University Shanghai Cancer Center, Shanghai, China

Clear cell renal cell carcinoma (ccRCC) constitutes the predominant subtype of renal cell carcinoma, representing roughly 75-80% of all instances.^1^^,^^2^ Among immune components in the TME, tumor-associated macrophages (TAMs) are a dominant population. Numerous studies have indicated that TAMs are closely linked to the onset and advancement of ccRCC.^3^^,^^4^ However, the potential of immunosuppressive TAMs as therapeutic targets warrants further investigation.

By removing the batch effect on the single-cell datasets, 7 samples and 45,290 cells were filtered after quality control. The top 20 principal components were selected for clustering and a total of 6 clusters were obtained (Supplementary Fig. 1A). Based on the cell markers, the clusters were annotated to a total of 4 cell subpopulations (Supplementary Fig. 1B). The cell markers and differential expression genes (DEGs) corroborated with the cell type identification (Supplementary Fig. 1C-E). A higher percentage of immune cells was detected in ccRCC patients than the percentage of other cell types (Supplementary Fig. 1F).

To understand the tumor immune environment in depth, the Immune_cells subpopulation was clustered, and grouped again (Supplementary Fig. 2A). Referring to the reported markers of immune cells^5^^,^^6^, we annotated immune cells into 7 cell types including (Supplementary Fig. 2B). Then, we identified the specific genes among the immune cell populations (Supplementary Fig. 2C-F) and focused on the macrophage population. Cluster 0 (Mac-0), cluster 7 (Mac-7), and cluster 8 (Mac-8) were identified as TAM. The functional enrichment analysis based on the specific genes validated the accuracy of cell type recognition (Supplementary Fig. 2G). Among all patients’ samples, macrophages and T cells constitute the main cellular components of the immune microenvironment in ccRCC (Supplementary Fig. 2H).

The TAM cell subpopulation was analyzed for differential gene expression with other immune cell subpopulations at single cell RNA-sequencing (scRNA-seq) level. A total of 240 DEGs were identified (Supplementary Fig. 3A and Supplementary Table 2). Next, we calculated the area under curve (AUC) values for distinguishing TAMs of these DEGs and screened the genes with AUC > 0.6 as TAM-specific genes. A total of 219 genes were identified, and two representative genes were shown here (Supplementary Fig. 3B and C, Supplementary Table 3). Functional enrichment analysis based on 219 TAM-specific genes revealed that these genes were significantly enriched in macrophage-associated functions such as macrophage activation, macrophage chemotaxis, macrophage migration, antigen processing and presentation, Fc-γ receptor-mediated phagocytosis, and phagosome (Supplementary Fig. 3D and Supplementary Table 4). In the FU-ccRCC-TKIs training cohort, univariate Cox and Lasso-Cox analyses of TAM-specific genes were performed, and eight prognostically significantly related genes were selected as TAM signature. Besides, we also compared a total of 50 TAM markers reported previously^5^^,^^7^^,^^8^ (Supplementary Table 5) with the 219 TAM-specific genes screened by us, and found three (*HLA-DRA, CD68, CD81*), three (*GPNMB, SLC40A1, MSR1*), and four (*MS4A6A, F13A, RNASE1, STAB1*) common genes, respectively (Supplementary Fig. 3E). However, none of the TAM signature genes were intersected with the above studies. Based on the FU-ccRCC-TKIs training cohort, the distribution of TAM signature in clinical features such as TAM score subgroups, TNM stage, International Society of Urological Pathology (ISUP) grade, and response (TKI treatment efficacy) was demonstrated. All TAM signature genes were significantly different in TAM score subgroups, and their expression was generally higher in the high TAM score group than in the low TAM score group, except for *EPB41L2* (Supplementary Fig. 3F). The different expression levels of TAM signature in the high TAM score group between the FU-ccRCC-TKIs training cohort and TCGA-KIRC validation cohort were demonstrated in Supplementary Fig. 3G. Then, the correlation of five sets of pro-cancer-related genes, including EMT, angiogenesis, TGF-β signaling pathway, autophagy, and fatty acid metabolism with TAM were analyzed. As shown in Supplementary Fig. 3H, genes such as *CD59* showed a significant positive correlation (*P*-value < 0.05) with most of the pro-cancer related genes. This implied that these genes may be involved in the regulation of the pro-cancer-related pathway, thus affecting tumor development. Supplementary Fig. 3I visualized Disabled-2 (DAB2), which was highly expressed in the high TAM score group at the bulk-RNA level, was also detected co-localized with CD68-labeled TAMs at the protein level.

Based on gene mutation data from the TCGA-KIRC cohort, the distribution of the top 20 mutated genes in TAM score subgroups was investigated. As shown in Supplementary Fig. 4A and B, genes such as *VHL* and *PBRM1* had high mutation frequencies in both the high TAM score and low TAM score groups. Fisher’s test comparing the mutation frequencies of patients in the TAM score subgroups revealed that patients in the low TAM score group had a significantly higher frequency of mutations in the *FREM2* gene, while patients in the high TAM score group had a higher frequency of mutations in the *ADAMTS12* gene (Supplementary Fig. 5A). Based on the known immunophenotyping information of TCGA-KIRC cohort, there was a significant difference in immunophenotyping distribution between TAM score subgroups (Supplementary Fig. 5B). Comparison of microsatellite instability (MSI) status among TAM score subgroups likewise revealed significant differences (Supplementary Fig. 5C). The TIMER algorithm was used to assess the differences in immune infiltration between TAM score subgroups, and it was found that in both the FU-ccRCC-TKIs training cohort and TCGA-KIRC validation cohort, CD4^+^ T cells and macrophages showed significant differences between TAM score subgroups (Supplementary Fig. 4C and Supplementary Fig. 5D). The expression levels of immune checkpoints such as HAVCR2 (also known as TIM-3) and LAG3 differed significantly between TAM score subgroups (Supplementary Fig. 4D and Supplementary. 5E), and the expression levels of HLA genes, such as *HLA-DQB1* and *HLA-DRA*, also differed significantly between TAM score subgroups (Supplementary Fig. 4E and Supplementary Fig. 5F). These observations indicated that the immune-exhaustion status and immune regulation were divergent between TAM score subgroups. Based on the TAM score grouping, the Kaplan-Meier curves showed a significant difference in survival between the two groups and a worse prognosis in the low TAM score group in both FU-ccRCC-TKIs training cohort and TCGA-KIRC validation cohort (Supplementary Fig. 4F and G). In addition, the ROC results showed that the AUCs of both the training and validation cohorts were above 0.66 and have better predictive efficacy than the TNM stage and ISUP grade (Supplementary Fig. 4H). The C-indexes of the TAM score model in both the training and validation cohorts were above 0.6, indicating that the model was stable (Supplementary Fig. 4I). The RMST ratio (high TAM score vs low TAM score) was greater than 1, which corroborated with the result that the low TAM score group predicted worse prognosis (Supplementary Fig. 4J). The results of univariate and multivariate Cox regression analysis confirmed that TAM score was an independent prognostic parameter (Supplementary Fig. 4K and Supplementary Table 6). This finding was validated in the TCGA-KIRC validation cohort (Supplementary Fig. 4L and Supplementary Table 7).

We collected proteomic data from 232 patients in our center for this study, namely the FU-ccRCC-proteome cohort, and explored the protein level differences of TAM signature between ccRCC tumor (T) and tumor-adjacent (TA) tissues. The results showed that all eight TAM signature genes differed significantly (Fig. 1A, upper panels). Subsequently, based on the protein expression levels of TAM signature in ccRCC patients, we divided the patients into high- and low-level groups based on the optimal breakpoints, and explored the prognostic differences of the patients between the high and low groups by Kaplan-Meier curves (Fig. 1A, lower panels). We found that DAB2 had low protein levels in ccRCC, and the prognosis was significantly worse in the DAB2 low-expression group. IHC staining validated that DAB2 was highly expressed in tumor-adjacent areas and less enriched in tumors. In addition, survival analysis of DAB2 expression in the FU-ccRCC-proteome cohort suggested that patients with DAB-high ccRCC had favorable prognosis (Fig. 1B). In order to explore the predictive value of the TAM score model in the proteome, we calculated TAM score based on our FU-ccRCC-proteome cohort using GSVA algorithm and TAM signature, and divided patients into high TAM score and low TAM score groups based on the median (Supplementary Table 8). As a result, there was a significant difference in patients’ prognosis between the TAM score subgroups at the protein level, and the prognosis of the low TAM score group was worse. The patients who reported dead were more clustered in the low TAM score group (Fig. 1C). To explore the predictive value of TAM score on TKI treatment efficacy, we illustrated the progression-free survival (PFS) differences between TAM score subgroups by Kaplan-Meier curves based on the FU-ccRCC-TKIs cohort. The results showed that the high TAM score group had a better prognosis, and that in patients with ccRCC, the high TAM score group may benefit more from immunotherapy (Fig. 1D). Similar results were seen in the external ccRCC immunotherapy cohort (Fig. 1E and Supplementary Table 9). Additionally, TAM score was calculated in the bladder cancer immunotherapy IMvigor210 cohort (Supplementary Table 10), and the Kaplan-Meier curves showed that there were significant differences in survival between TAM score subgroups. However, the low TAM score group had a better prognosis and objective response to treatment (Supplementary Fig. 6A). This suggested that in bladder cancer patients, the low TAM score group may also benefit more from immunotherapy (Supplementary Fig. 6B-D).

**Fig. 1:**
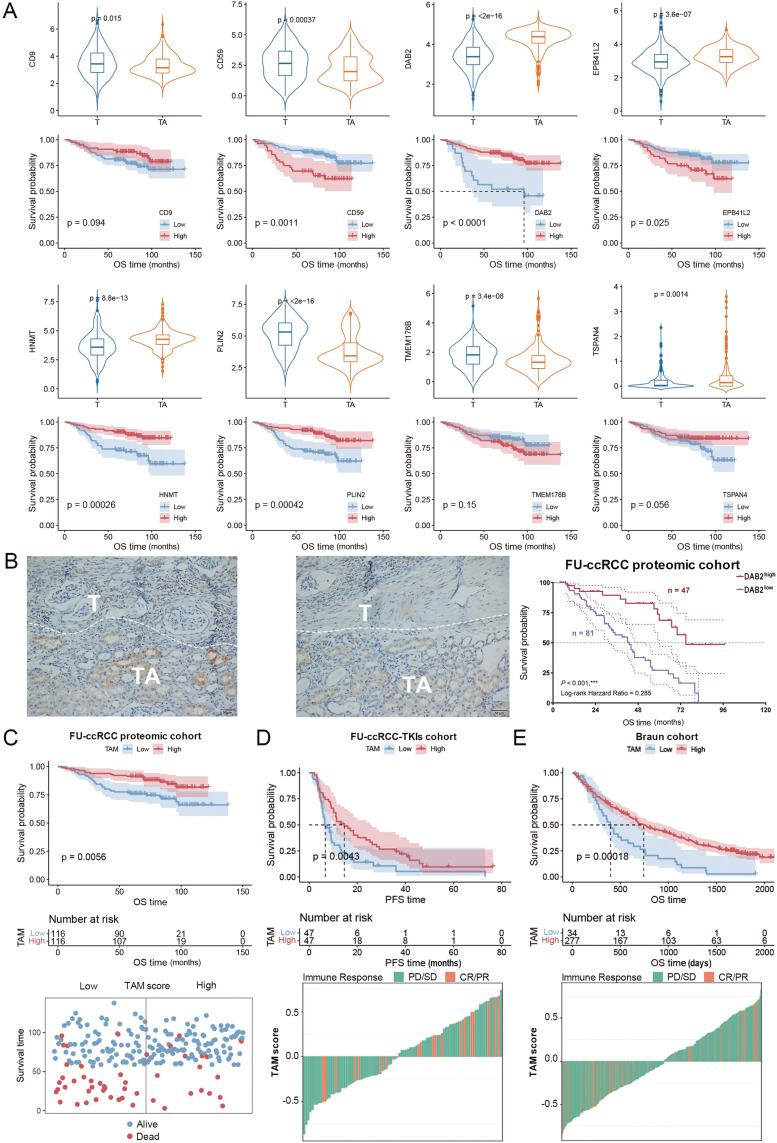
Prediction value of TAM signature and TAM score at protein level. (A) Differences in protein levels of TAM signature between tumor (T) and tumor-adjacent (TA) tissues (top), and prognostic differences between high and low TAM score subgroups in patients (bottom) from the FU-ccRCC-proteome cohort. (B) Representative immunohistochemistry staining of DAB2 in tumor (T) and tumor-adjacent (TA) tissues (left), and survival analysis of DAB2 expression in the FU-ccRCC-proteome cohort (right). Scale bar, 50 μm. (C) Kaplan–Meier curves of OS in high and low TAM score subgroups (top), and TAM score distribution (bottom) in patients from the FU-ccRCC-proteome cohort. (D) Kaplan–Meier curves of PFS (top), and distribution of immune response to TKI treatment with TAM score (bottom) in the FU-ccRCC-TKIs cohort. (E) Kaplan–Meier curves of OS (top), and distribution of immune response to TKI treatment with TAM score (bottom) in the Braun cohort. ccRCC, clear cell renal cell carcinoma; CR, complete response; DAB2, Disabled-2; OS, overall survival; PD, progressive disease; PFS, progression-free survival; PR, partial response; SD, stable disease; T, tumor; TA, tumor-adjacent; TAM, tumor-associated macrophage; TKI, tyrosine kinase inhibitor.

To explore the molecular basis of the TAM signature, we selected one of the 8 TAM signature genes, *PLIN2*, as a target. We used lentiviral technique to construct PLIN2 knockdown (KO) macrophages, and induced PLIN2 expression and over-expression (OE) in macrophages derived from THP-1 cells (Supplementary Fig. 7A). PLIN2^+^ macrophages producing IL-1β, IL-6 and TNF-α, suggesting its pro-inflammatory phenotype, while the production of such cytokines in PLIN2-KO macrophages was significantly less (Supplementary Fig. 7B). Co-culture of macrophages with ccRCC cell lines suggested that knockdown of PLIN2 in macrophages significantly promoted the growth of tumor cells, though over-expressing of PLIN2 did not help with tumor-suppression (Supplementary Fig. 7C). The wound healing assay suggested that the migration and invasion ability of tumor cells were suppressed by PLIN2^+^ macrophages (Supplementary Fig. 7D). Interestingly, flow cytometry examination of PLIN2^+^ macrophages expressed low-levels of PD-L1, CD206, and CD163, while PLIN2-KO macrophages showed high-levels of these receptors (Supplementary Fig. 7E). Multispectral imaging of multiple IF-stained ccRCC samples illustrated that PLIN2 is highly expressed in CD68^+^ macrophages but not in CD163^+^ macrophages (Supplementary Fig. 7F), demonstrating significant spatial colocalization of PLIN2 and CD68^+^CD163^-^ M1 macrophages in ccRCC tissues. This observation corroborated with the flow cytometry data, indicating that PLIN2^+^ TAMs may not be classified as the M2-like macrophages.

In summary, using scRNA-seq and bulk RNA-seq, we analyzed the ccRCC microenvironment, with a focus on TAM heterogeneity and function. Immune cells, particularly macrophages, were enriched in ccRCC and exhibited distinct gene expression profiles. Bulk RNA-seq validated high TAM infiltration in advanced tumors, and an 8-gene TAM signature was identified. This signature correlated with tumor progression, recurrence, and survival, and served as a predictive marker for ICI response. Among these genes, PLIN2 was associated with pro-inflammatory TAM phenotypes and better prognosis. Functional assays confirmed its role in cytokine secretion in co-cultures with ccRCC cells.

## CRediT authorship contribution statement

**Jiahe Lu:** Conceptualization, Data curation, Formal analysis, Funding acquisition, Investigation, Methodology, Validation, Writing – original draft, Writing – review & editing. **Shiqi Ye:** Conceptualization, Data curation, Formal analysis, Investigation, Methodology, Resources, Software, Validation, Writing – original draft. **Zhongyuan Wang:** Investigation, Methodology, Writing – original draft, Writing – review & editing. **Yunhong Zhou:** Methodology, Validation, Formal analysis, Resources, Data curation, Writing – review & editing, Visualization. **Aihetaimujiang Anwaier:** Investigation, Methodology, Validation. **Siqi Zhou:** Data curation, Formal analysis, Resources, Software. **Kun Chang:** Validation, Writing – review & editing. **Hailiang Zhang:** Funding acquisition, Resources, Software, Supervision, Writing – review & editing. **Dingwei Ye:** Funding acquisition, Resources, Software, Supervision, Writing – review & editing. **Wenhao Xu:** Conceptualization, Data curation, Formal analysis, Funding acquisition, Investigation, Methodology, Resources, Software, Supervision, Validation, Writing – original draft, Writing – review & editing.

## Appendix Supplementary materials

Image, application 1

Image, application 2
