# Supplementary material for: Stratification system with tumor-associated macrophages for predicting prognostic and therapeutic implications in clear cell renal cell carcinoma
Source: J Natl Cancer Cent. 2026 Apr 24;6(3):316–9. doi: 10.1016/j.jncc.2024.12.009 (PMC13250520; doi:10.1016/j.jncc.2024.12.009)
Supplement: Supplementary file 1 [file mmc1.pdf]

Supplementary materials

Stratification system with tumor-associated macrophages for predicting prognostic and therapeutic implications in clear cell renal cell carcinoma

Jiahe Lu[1,2,†], Shiqi Ye[1,2,†], Zhongyuan Wang[1,2,†], Aihetaimujiang Anwaier[1,2], Siqi Zhou[1,2], Kun Chang[1,2], Hailiang Zhang[1,2,*], Dingwei Ye[1,2,*], Wenhao Xu[1,2,*]

Materials and methods

Collection of expression profiles and clinical data

In this study, we acquired and processed two clinical cohorts' data from Fudan University Shanghai Cancer Center (FUSCC), including FU-ccRCC-TKIs (bulk RNA-seq profiles and clinical data of 94 ccRCC tissues received tyrosine kinase inhibitor [TKIs] treatment) and FU-ccRCC-proteome (proteomic profiles and clinical data of 232 ccRCC and adjacent normal tissues). We employed the R package "TCGAbiolinks" to download the bulk RNA-seq profiles and clinical information of the TCGA-KIRC dataset (524 ccRCC tissues) as validation set. Furthermore, we collected ccRCC single-cell datasets GSE152938 and GSE156632 from the GEO database (https://www.ncbi.nlm.nih.gov/geo). In the GSE152938 dataset, we excluded control, papillary RCC (pRCC) and chromophobe RCC (chRCC) samples, and retained 2 ccRCC samples (GSM4630028 and GSM4630029). In the GSE156632 dataset, we excluded control and mixed samples, retaining 5 ccRCC samples: GSM4735364, GSM4735366, GSM4735368, GSM4735372, and GSM4735374. Besides, we downloaded a ccRCC immunotherapy cohort (Braun cohort) with comprehensive bulk RNA-seq profiles, survival data, and immunotherapy response information of 281 samples[1]. An additional immunotherapy cohort (IMvigor210 cohort) of 298 bladder cancer patients was obtained by the R package 'IMvigor210CoreBiologies'. The detailed baseline data of the cohorts was summarized in Supplementary Table 11.

Processing of scRNA-seq data

The two scRNA-seq datasets were preprocessed with the R package Seurat (v4.1.0). To exclude low-quality cells and genes with rare low expression, the following thresholds were applied: (1) Each gene must be expressed in at least 3 cells; (2) For each cell, the feature and counts were restricted to the range of 2.5% to 97.5%; (3)

Mitochondrial genes constituted less than 20% of the whole gene expression. Next, we merged the two datasets using the 'merge' algorithm and performed data normalization with the 'NormalizeData' algorithm. The top 4500 highly variable genes were identified by 'FindVariableFeatures' algorithm. The 'RunHarmony' algorithm was conducted to correct the batch effects and mitigate downstream analysis interference. Subsequently, the data underwent scaling transformation, and principal component analysis (PCA) was employed for dimensionality reduction, with the top 20 principal components selected for downstream analysis. The clustering analysis was performed with the resolution = 0.01, and uniform manifold approximation and projection (UMAP) was applied for dimensionality reduction and visualization of the cell clusters. The 'FindAllMarkers' algorithm was applied to identify specific genes for each cluster with log fold change threshold set at 0.25 and a minimum percentage of 0.1 for cell type markers. By referring to cell markers reported by Su C et al.[2] and Zhang M et al.[3] , we annotated the clusters and ultimately determined four distinct cell types: endothelial cells, epithelial cells, fibroblasts, and immune cells.

Characterization of immune fractions

The data of immune cells subset was normalized, standardized, and removed batch effect and the top 4500 variable genes were hunted for principal component analysis (PCA). Then, the clustering and subgrouping of immune cells were conducted again. Referring to immune cell markers reported by Su C et al.[2] and Zhang M et al.[3], we annotated the immune cell clusters and ultimately determined seven distinct immune cell types: macrophage, monocyt, T cell, NK cell, B cell, dendritic cell (DC), and mast cell. Furthermore, we identified the tumor-associated macrophage (TAM) according to the classic markers reported by Su C et al.[2] and visualized the expression levels of the markers by VlnPlot. The 'FindAllMarkers' algorithm was applied to identify the specific genes among immune cell subgroups (avg_log2F > 1 and *P*_val_ad < 0.05), and enrichment analyses were performed by R package 'clusterProfiler'.

Analyzation of macrophage infiltration level

The infiltrating fractions of M0-macrophage, M1-macrophage, and M2-macrophage were identified with the single-sample gene set enrichment analysis (ssGSEA) algorithm in R. The R package 'ggpubr' and 'pheatmap' were applied to visualize the infiltration level of different macrophage subgroups. The association of TAM infiltration level and TNM (tumor [T], node [N], and distant metastasis [M]) stage

and International Society of Urological Pathology (ISUP) grade was analyzed by R package 'ggpubr'.

Identification of TAM gene markers

Differential expression genes (DEGs) among TAM subgroup and other cell subgroups were identified with the "Seurat" package in R software, with the criteria of avg_log2fc > 0.585 and *p*_val_adj < 0.05. The area under the curves (AUCs) of DEGs was calculated with "pROC" package, and DEGs with an AUC > 0.6 were defined as TAM gene markers. The function enrichment analysis was conducted by "clusterProfiler" package with the gene markers.

Construction of TAM signature

To validate the robustness of the TAM gene markers, we applied FU-ccRCC-TKIs cohort as the training cohort, and TCGA-KIRC as the validation cohort to construct a TAM signature. We first performed the univariate Cox regression analysis with the expression of each TAM gene marker. Then, LASSO-Cox algorithm in 'glmnet' R package was conducted to determine prognosis-related genes as the TAM signature genes. The TAM score was calculated with the signature genes by gene set variation analysis (GSVA) algorithm (parameters method = 'gsva', kcdf = 'Gaussian'), the TAM signature is used as the feature gene set, and the TAM signature enrichment score for each sample is computed based on the expression spectrum of the training cohort. Patients were stratified into low and high TAM score groups by the median TAM score.

Evaluation of the clinical correlation of the TAM signature

We first analyzed the differences in TNM stage, ISUP grade, and immunotherapy response between the high and low TAM score groups. Then, we analyzed the correlations between five cancer-promoting related gene sets, including epithelial-mesenchymal transition (EMT), angiogenesis, TGF-β signaling pathway, autophagy, and fatty acid metabolism, with the TAM signature by R package 'circlize'. The EMT gene set was obtained from the dbEMT2 database (http://dbemt.bioinfo-minzhao.org/index.html), the TGF-β signaling pathway gene set was obtained from the HALLMARK_TGF_BETA_SIGNALING of the MSigDB database (https://www.gsea-msigdb.org/gsea/msigdb), the angiogenesis gene set was obtained from the HALLMARK_ANGIOGENESIS of MSigDB database, the autophagy gene set was obtained from the HADb database (http://www.autophagy.lu/index.html), and

fatty acid metabolism gene set was obtained from HALLMARK_FATTY_ACID_METABOLISM and KEGG_FATTY_ACID_METABOLISM of HALLMARK_FATTY_ACID_ of MSigDB database

Analyzation of the immune landscape in TAM signature

The top 20 most frequently mutated genes in high and low TAM score groups were displayed with 'maftools' R package. The tumor mutation burden (TMB) and homologous recombination deficiency (HRD) information of ccRCC patients in TCGA-KIRC cohort were downloaded from the work of Knijnenburg et al.[4] The neoantigen load of ccRCC patients in TCGA-KIRC cohort was downloaded from the work of Thorsson et al.[5] The microsatellite instability (MSI) status of ccRCC patients in TCGA-KIRC cohort was downloaded from the work of Li et al.[47] The immune phenotyping of ccRCC patients in TCGA-KIRC cohort was downloaded from UCSC XENA database (https://tcga-pancan-atlas-hub.s3.us-east-1.amazonaws.com/download/Subtype_Immune_Model_Based.txt.gz). The TIMER algorithm was applied to evaluate the infiltration fraction of immune cells and expression level of immune checkpoints.

Prediction of the survival outcomes and treatment response with TAM signature

The survival difference between the two PRMTGroups was evaluated using the Kaplan–Meier analysis. The receiver operating characteristic (ROC) curve of TAM score was constructed by the 'pROC' R package. The C-index was calculated with the 'Hmisc' R package, and the restricted mean survival time (RMST) ratio was calculated with the 'survRM2' R package. Furthermore, FU-ccRCC-TKIs cohort was used to validate the predictive power of the TAM score to TKI treatment response with 'pROC' R package. The Braun cohort and IMvigor210 cohort were applied to verify the predictive power of the signature to immunotherapy response.

Validation of TAM signature at the protein level

Based on the FU-ccRCC-proteome data, the protein expression levels of TAM signature genes were displayed by violin plot with 'ggpubr' R package. The survival difference between patients group stratified by distinct TAM signature genes was analyzed by Kaplan–Meier analysis. Then, the TAM score of each ccRCC patient was

calculated by ‘GSVA’ R package, and patients were stratified into low and high TAM score groups by the median TAM score. The Kaplan–Meier analysis and ROC analysis were carried out to assess the predictive efficacy of the TAM signature.

Immunohistochemistry and multiplex immunofluorescence staining assays

Immunohistochemistry (IHC) staining assay was conducted to evaluate the expression difference of disabled-2 (encoded by *DAB2*) in ccRCC and tumor-adjacent tissues from FUSCC following manufacturers’ protocols as previously described[33]. Deparaffinized sections were incubated with primary anti- disabled-2 antibody (1:1000, #12906, CST) at 4 °C overnight, and then incubated species-appropriate secondary antibody. The images were taken with an inverted microscope (Olympus, Tokyo, Japan). The immunofluorescence (mIF) staining assay was carried out to assess the distribution of disabled-2 (1:1600) or perilipin-2 (encoded by *PLIN2*) (1:100, #79979, CST), CD68 (1:1000, ab213363, Abcam), CD163 (1:500, ab182422, Abcam), cytokeratin (CK) (1:100, ab7753, Abcam) and DAPI (1:2000, #4083, CST) in ccRCC tissues following manufacturers’ protocols as previously described[33]. Multiplex stained slides were scanned using a Vectra polaris quantitative pathology imaging system (Akoya Biosciences). CaseViewer software was applied to visualize the images.

Co-cultures of macrophages with RCC cell lines

THP-1 cells were co-cultured with 786-O and CAKI-1 tumor cells using a Transwell culture system equipped with 0.4 μm pore polyester membrane inserts (Corning HTS Transwell, Corning, USA) for a period of three days. In the macrophage-focused experiments, THP-1 cells were plated in 6-well plates at a density of 1×10^5 cells per well. 786-O and CAKI-1 cells were seeded at 4×10^5 cells per well in the Transwell inserts. For the tumor cell-focused experiments, 786-O and CAKI-1 cells were seeded directly onto the well plates, while THP-1 cells were placed in the Transwell chambers.

Transfection

Lentiviral constructs for PLIN2 knockdown or overexpression were acquired from OBIO (Obio Technology Corp, China). Cells were seeded in 6-well plates at 50% confluence and then infected with either PLIN2 overexpression lentivirus, a negative control, PLIN2 knockdown lentivirus, or a scrambled control, respectively. Stable

transduced cell pools were established through selection with puromycin (4 μg/ml) over a period of two weeks. Transfections were carried out using the Lipofectamine 3000 reagent (Invitrogen) following the manufacturer's guidelines.

Protein extraction and western blot

Total proteins from cells were isolated using RIPA lysis buffer (Sigma). The concentration of the extracted proteins was then quantified with a BCA protein assay kit (Beyotime, China). Protein samples were separated by SDS-PAGE and subsequently transferred to polyvinylidene fluoride (PVDF) membranes. These membranes were blocked with 5% non-fat milk to prevent non-specific binding, followed by incubation with specific primary antibodies and corresponding secondary antibodies (Proteintech, China). After thorough washing, the presence of target proteins was visualized via chemiluminescence detection (Bio-Rad), and the resulting blots were analyzed using Image Lab software (Bio-Rad).

Enzyme-linked immunosorbent assay

Enzyme-linked immunosorbent assay (ELISA) kits were utilized to measure the levels of IL-1β, TNF-α, and IL-6 in the supernatants from macrophage cultures. After centrifuging the culture supernatants at room temperature to remove the cells, the relative concentrations of IL-1β, TNF-α, and IL-6 in the supernatants were determined using ELISA.

Transwell assay

For the transwell assay, co-cultured 786-0 and CAKI-1 cells were seeded into the upper chambers, which were either coated with Matrigel (BD Biosciences, USA) or left uncoated, using serum-free medium. The lower chambers were filled with medium containing 10% FBS. Following a 24-hour incubation at 37°C, the cells remaining on the upper surface of the membrane were gently removed using cotton swabs. The cells that had migrated to the lower surface of the membrane were fixed with 10% formalin and stained with crystal violet for 15 minutes at room temperature. Finally, the number of migrated cells was quantified.

Wound scratch assay

To evaluate the impact of co-cultured system on cell migration, a wound scratch

assay was performed. When the transfected cells in 6-well plates reached 90-95% confluence, a linear scratch was created using a 200-µl pipette tip. The wells were then rinsed with phosphate-buffered saline (PBS) to eliminate any dislodged cells. The cultures were maintained at 37°C in a humidified incubator with 5% CO2. Wound closure was documented at 0- and 24-hours post-scratch using a digital camera system (Olympus Corp, Japan).

Cell counting kit-8 assay

To assess cell proliferation, cells were plated in 96-well plates at a density of 2000 cells per well. Cell viability was evaluated at 0, 24-, 48-, 72-, and 96-hours post-seeding using the Cell counting kit-8 (CCK-8) assay (Dojindo, Japan), following the manufacturer's protocol. Specifically, 10 µL of CCK-8 solution was added to each well, and the plate was incubated for 1 hour at 37 °C in the dark. Subsequently, the absorbance was read at 450 nm using a microplate reader (Tecan, Switzerland).

Flow cytometry

Flow cytometry was utilized to determine macrophage polarization following the manufacturer's guidelines. To identify macrophages, we employed CD68 antibody (14-0681-82, Invitrogen). For the detection of M1 macrophages, CD86 antibody (12-0862-81, Invitrogen) was used. For the detection of M2 macrophages, CD206 (17-2061-80, Invitrogen), CD163 (17-1631-80, Invitrogen) and CD274 (12-5982-81, Invitrogen) were used.

References

1. Braun DA, Hou Y, Bakouny Z, et al. Interplay of somatic alterations and immune infiltration modulates response to PD-1 blockade in advanced clear cell renal cell carcinoma. *Nat Med*. 2020;26(6):909-918.

2. Su C, Lv Y, Lu W, et al. Single-cell RNA sequencing in multiple pathologic types of renal cell carcinoma revealed novel potential tumor-specific markers. *Front Oncol*. 2021;11:719564.

3. Zhang M, Zhai W, Miao J, et al. Single cell analysis reveals intra-tumour heterogeneity, microenvironment and potential diagnosis markers for clear cell renal cell carcinoma. *Clin Transl Med*. 2022;12(5):e713.

4. Knijnenburg TA, Wang L, Zimmermann MT, et al. Genomic and Molecular Landscape of DNA

Damage Repair Deficiency across The Cancer Genome Atlas. *Cell Rep*. 2018;23(1):239-254.e6. doi:10.1016/j.celrep.2018.03.076

5. Malta TM, Noushmehr H, Network CGAR, others. The immune landscape of cancer. *Immunity*. 2018;48(4):812-830.

Figure legends

Supplementary Fig. 1. Tumor microenvironment landscape of clear cell renal cell carcinoma. (A) UMAP of cell clustering from renal cell carcinoma based on single-cell RNA-sequencing data. (B) UMAP of annotated cell subpopulations in renal cell carcinoma dataset. (C) Bubble chart of the expression levels of cell markers in different cell subpopulations. (D) UMAP of the expression of indicated cell markers in different cell subpopulation clusters. (E) Heatmap of top 3 differentially expressed genes in different cell subpopulations. (F) The proportion of different cell subpopulations in indicated renal cell carcinoma patients. UAMP, uniform manifold approximation and projection.

Supplementary Fig. 2. Tumor microenvironment landscape of ccRCC. (A) UMAP of cell clustering from the immune cell subpopulation. (B) UMAP of annotated immune cell subpopulations. (C) Violin plot of the expression level of indicated TAM markers in four different prospective macrophage clusters. (D) Bubble chart of the expression levels of immune cell markers in different immune cell subpopulations. (E) Heatmap of top 3 differentially expressed genes in different immune cell subpopulations. (F) UMAP of the distribution of indicated immune cell markers in immune cell clusters. (G) Functional enrichment of specific genes of each immune cell subpopulation. (H) Proportion of different immune cell subpopulations in indicated ccRCC patients. ccRCC, clear cell renal cell carcinoma; TAM, tumor-associated macrophage; UAMP, uniform manifold approximation and projection.

Supplementary Fig. 3. Screening of TAM-specific genes and TAM signature. (A) Volcano plot of differentially expressed genes in TAMs compared to other immune cell subpopulations. (B) Heatmap of the expression of TAM-specific genes that distinguished TAMs from other immune cell subpopulations. (C) ROC curves for C1QA and C1QB. (D) Functional enrichment of 219 TAM-specific genes. (E) Venn diagram of the intersection of TAM markers screened above and collected by other studies. (F) Heatmap of TAM signature gene expression in TAM score subgroups, TNM stage, ISUP grade, and response. Asterisk stands for statistical significance; *, $P < 0.05$; **, $P < 0.01$; ***, $P < 0.001$; ****, $P < 0.0001$. (G) Bubble chart of TAM signature gene expression in high TAM score subgroup in FU-ccRCC-TKIs and TCGA-KIRC cohorts. (H) Heatmap of the correlation of TAM signature gene expression with epithelial–mesenchymal transition, angiogenesis, TGF-β signaling pathway, autophagy, and fatty acid metabolism pathways. (I) Multiplex immunofluorescence images of

ccRCC sample. Scale bars, 20μm (top) and 50 μm (bottom). AUC, area under curve; ccRCC, clear cell renal cell carcinoma; CK, cytokeratin; CR, complete response; DAB2, Disabled-2; FC, fold change; ISUP, International Society of Urological Pathology; NS, non-significant; PD, progressive disease; PR, partial response; ROC, Receiver operating characteristic; SD, stable disease; TAM, tumor-associated macrophage.

Supplementary Fig. 4. TAM score correlates with survival time of patients with ccRCC. (A) Waterfall diagram of the top 20 mutated genes in high TAM score group. (B) Waterfall diagram of the top 20 mutated genes in low TAM score group. (C) Infiltration of different immune cell types in TAM score subgroups in FU-ccRCC-TKIs cohort. (D) Expression of different human leukocyte antigen genes in TAM score subgroups in FU-ccRCC-TKIs cohort. (E) Expression of different immune checkpoints in TAM score subgroups in TCGA-KIRC cohort. (F) Kaplan-Meier curves analyzed differences in OS for patients with high and low TAM score from FU-ccRCC-TKIs cohort. (G) Kaplan-Meier curves analyzed differences in OS for patients with high and low TAM score from TCGA-KIRC cohort. (H) Receiver operating characteristic curves of TAM score in FU-ccRCC-TKIs cohort and TCGA-KIRC cohort, TNM stage and ISUP grade. (I) C-index of the TAM score model in FU-ccRCC-TKIs cohort and TCGA-KIRC cohort. (J) RMST ratio of the TAM score model in FU-ccRCC-TKIs cohort and TCGA-KIRC cohort. (K) Univariate and multivariate Cox regression analysis identifying independent prognostic parameters in FU-ccRCC-TKIs cohort. (L) Univariate and multivariate Cox regression analysis identifying independent prognostic parameters in TCGA-KIRC cohort. Asterisk stands for statistical significance, *, $P < 0.05$; **, $P < 0.01$; ***, $P < 0.001$; ****, $P < 0.0001$. ccRCC, clear cell renal cell carcinoma; CI, confidence interval; ISUP, International Society of Urological Pathology; OS, overall survival; RMST, restricted mean survival time; TAM, tumor-associated macrophage.

Supplementary Fig. 5. Portraying of the immunological features of the TAM score subgroups. (A) Forest plot of the mutation frequency in TAM score high and low groups. (B) The proportion of immunophenotyping distribution in two TAM score subgroups. (C) The proportion of microsatellite instable status in two TAM score subgroups. (D) Infiltration of different immune cell typesin TAM score subgroups in TCGA-KIRC cohort. (E) Expression of different immune checkpoints in TAM score subgroups in TCGA-KIRC cohort. (F) Expression of different HLA genes in TAM score subgroups in TCGA-KIRC cohort. Asterisk stands for statistical significance; *, $P < 0.05$; **, $P < 0.01$; ***, $P < 0.001$; ****, $P < 0.0001$. HLA, human leukocyte antigen; MSI, microsatellite instable; MSS, microsatellite stable; TAM, tumor-

associated macrophage.

Supplementary Fig. 6. Prediction value of TAM score in bladder cancer. (A) Kaplan–Meier curves of PFS of TAM score subgroups in IMvigor210 cohort. (B) Distribution of immune response to TKI treatment in TAM score subgroups in IMvigor210 cohort. (C) Box-line plot of differences in TAM score among patients with different immune response to TKIs treatment. (D) Distribution of immune response to TKI treatment with TAM score in IMvigor210 cohort. CR, complete response; OS, overall survival; PD, progressive disease; PR, partial response; SD, stable disease; TAM, tumor-associated macrophage; TKIs, tyrosine kinase inhibitors.

Supplementary Fig. 7. $PLIN2^+$ macrophages shows a pro-inflammatory phenotype. (A) Western blotting of PLIN2-normal expressing, OE and KO in macrophages derived from THP-1 cells. (B) Cytokine expression by PLIN2-normal expressing, OE and KO macrophages assessed by ELISA. (C) Cell invasion and proliferation of ccRCC cell lines co-culture with PLIN2-normal expressing, OE and KO macrophages. (D) Wound healing assay of ccRCC cell lines co-cultured with PLIN2-normal expressing, OE and KO macrophages. (E) Flow cytometry assessment of CD274 (PD-L1), CD206, and CD163 expression on PLIN2-normal expressing, OE and KO macrophages. (F) Multiplex immunofluorescence images of ccRCC samples. White arrows indicate the representative $PLIN2^+$ macrophages. Scale bars, 50 μm. ccRCC, clear cell renal cell carcinoma; KO, knocked-down; NC, negative control; OE, over expressing; PLIN2, Perilipin-2; SH, shRNA.

Supplementary Fig. 1

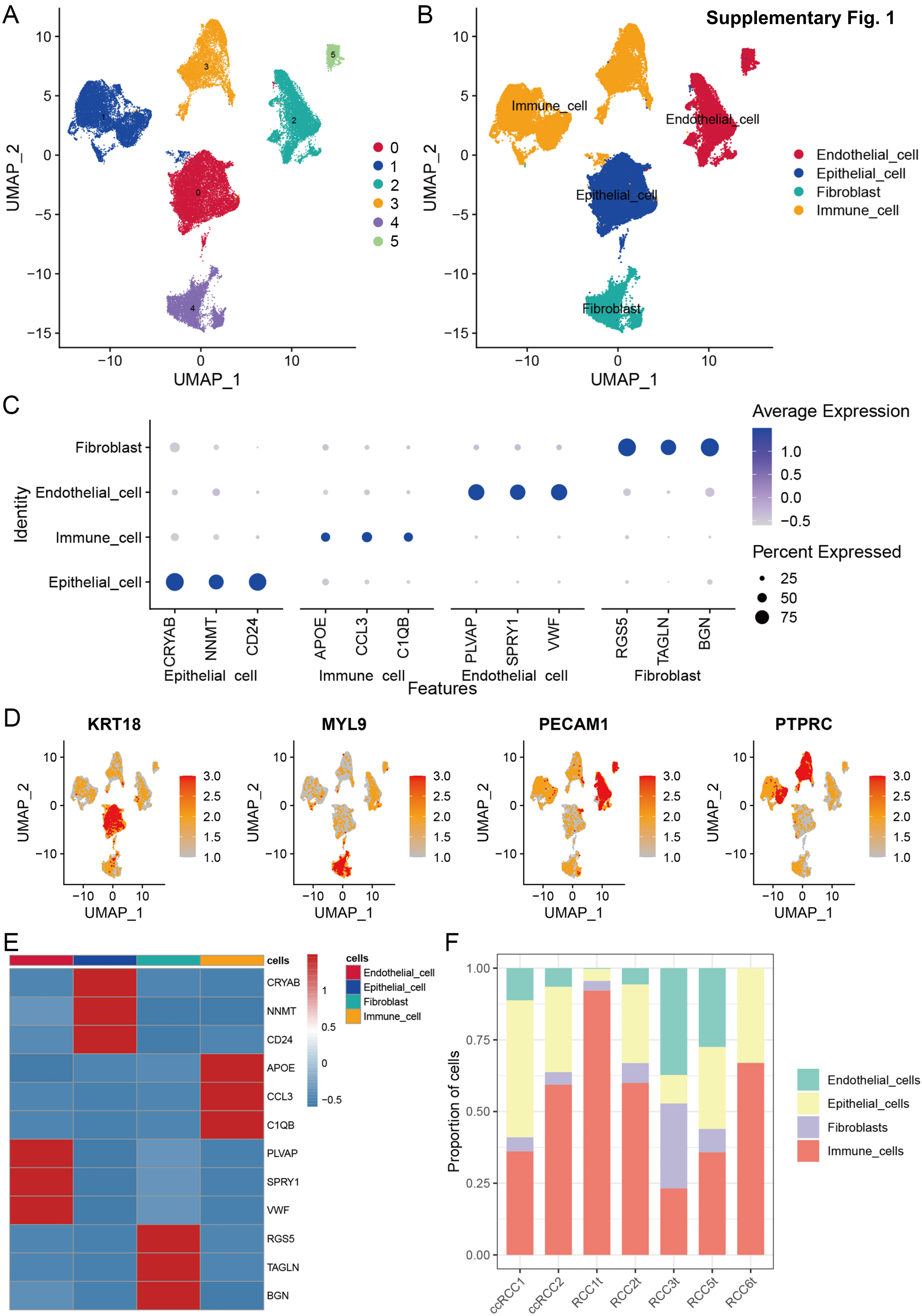

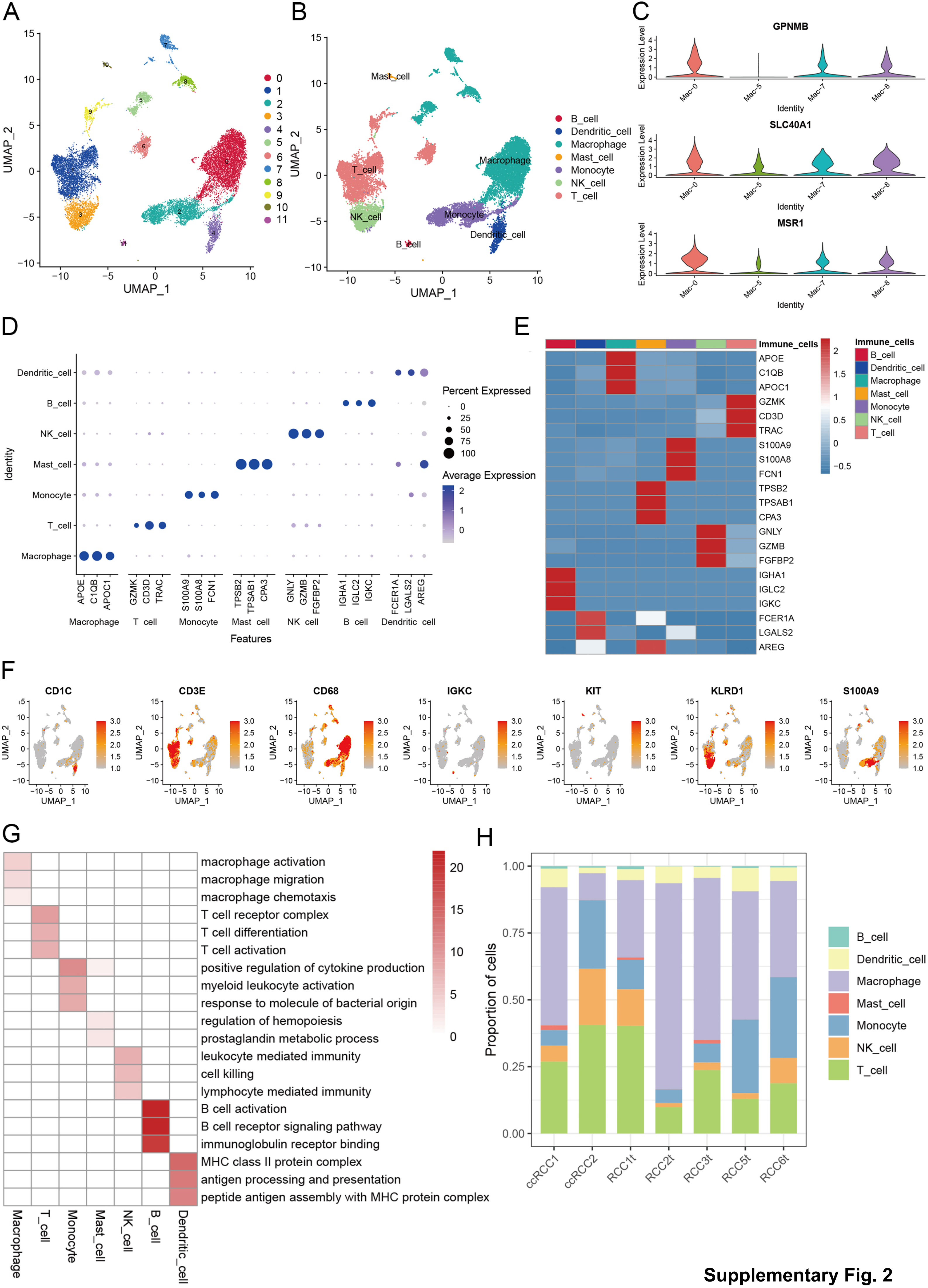

Supplementary Fig. 2

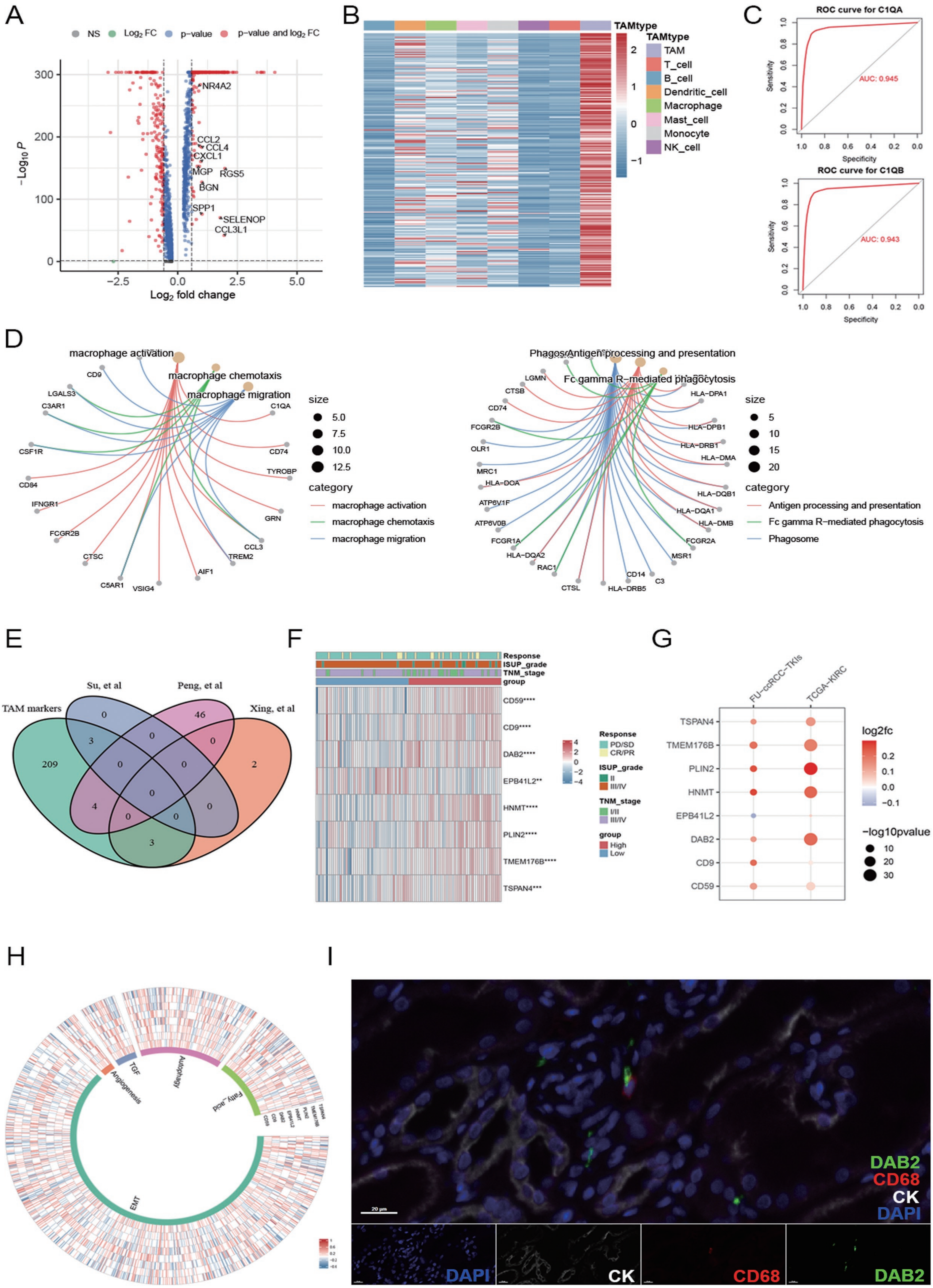

Supplementary Fig. 3

Supplementary Fig. 4

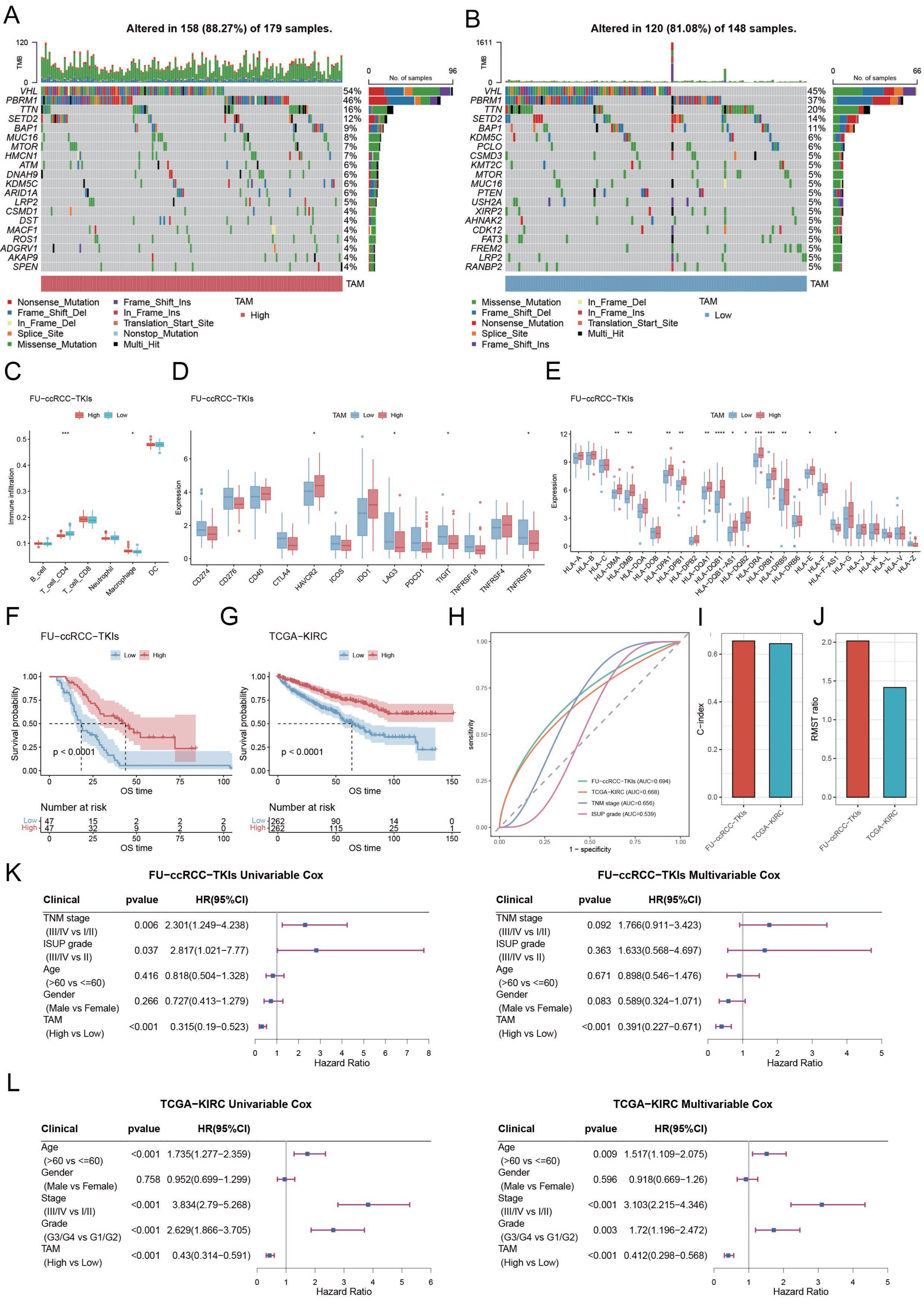

Supplementary Fig. 5

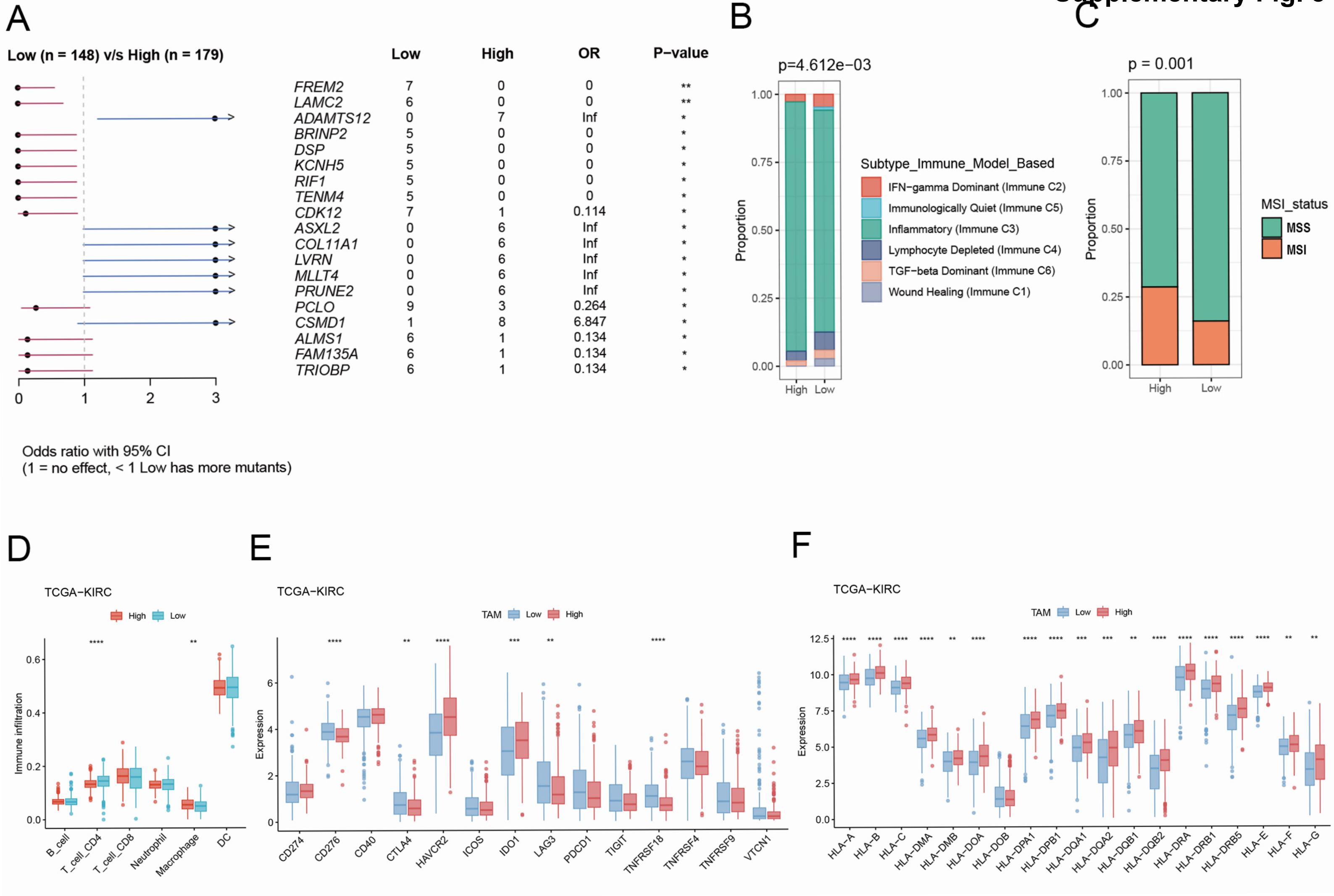

Supplementary Fig. 6

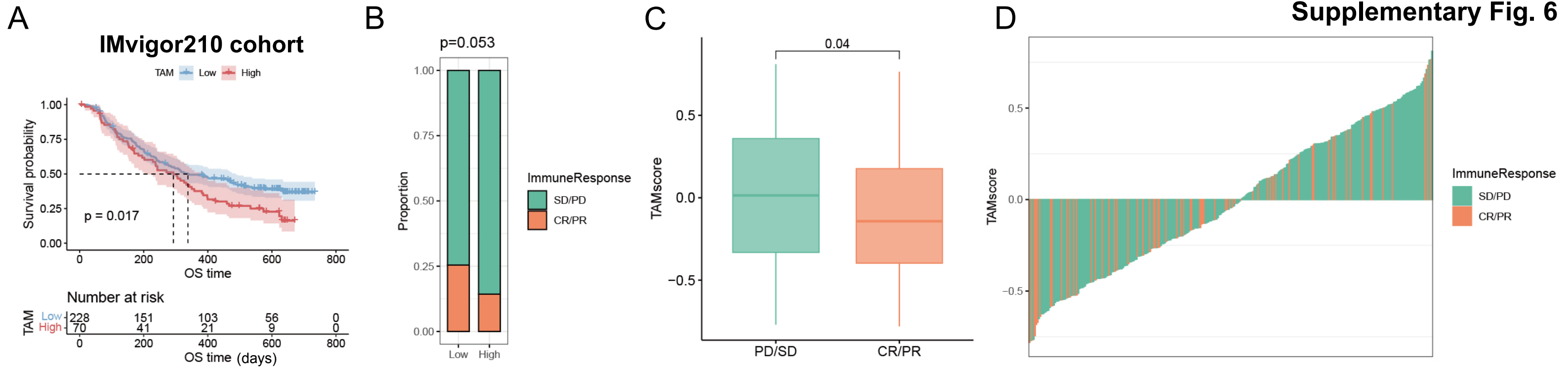

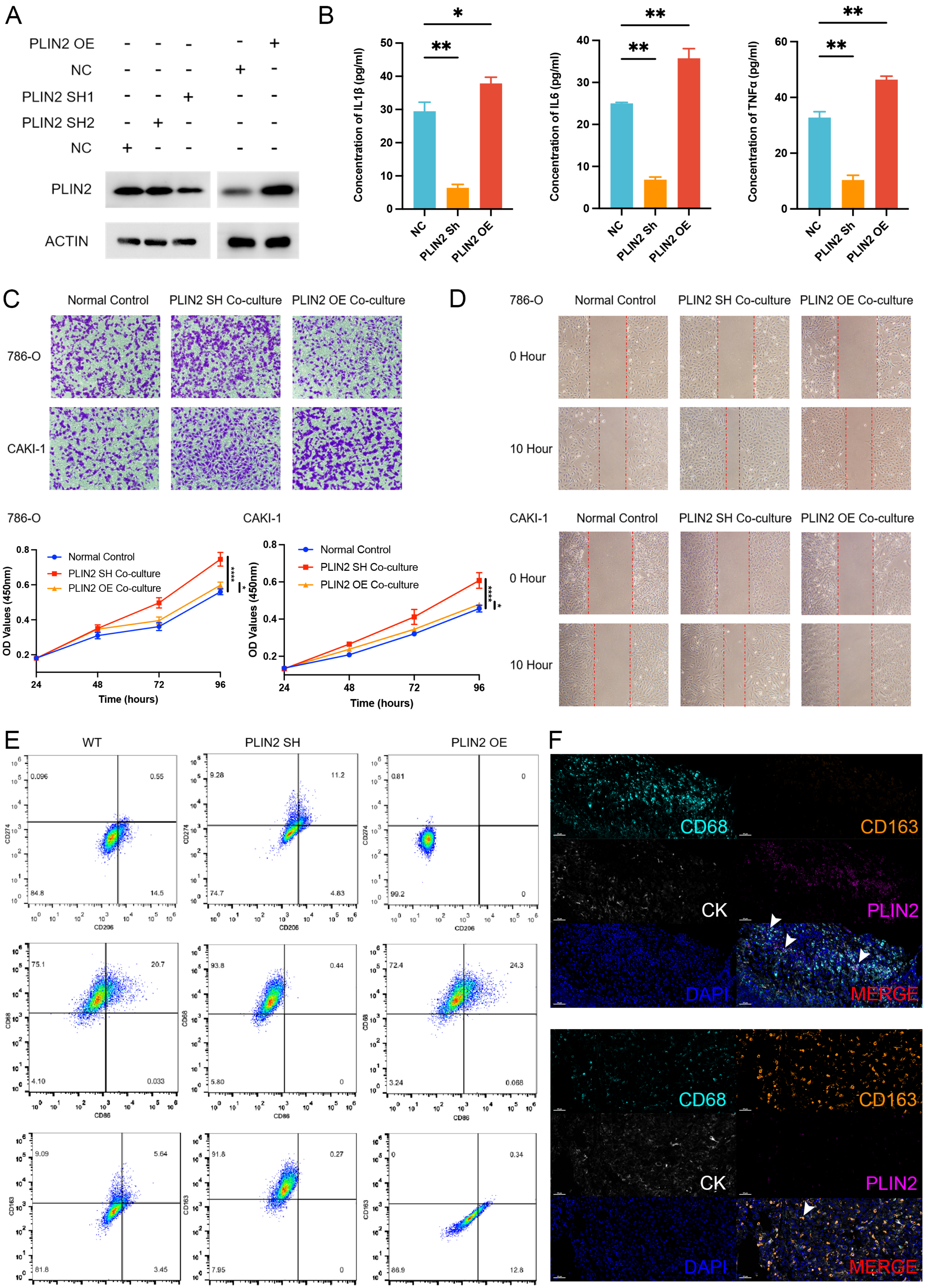

Supplementary Fig. 7
